# Diagnostic Accuracy of Machine Learning-Based Radiomics in Grading Gliomas: Systematic Review and Meta-Analysis

**DOI:** 10.1155/2020/2127062

**Published:** 2020-12-18

**Authors:** Curtis K. Sohn, Sotirios Bisdas

**Affiliations:** ^1^Queen Square Institute of Neurology, University College London, Queen Square 7, London WC1N 3BG, UK; ^2^Lysholm Department of Neuroradiology, The National Hospital for Neurology and Neurosurgery, Queen Square 8-11, London WC1N 3BG, UK

## Abstract

**Purpose:**

This study aimed to estimate the diagnostic accuracy of machine learning- (ML-) based radiomics in differentiating high-grade gliomas (HGG) from low-grade gliomas (LGG) and to identify potential covariates that could affect the diagnostic accuracy of ML-based radiomic analysis in classifying gliomas.

**Method:**

A primary literature search of the PubMed database was conducted to find all related literatures in English between January 1, 2009, and May 1, 2020, with combining synonyms for “machine learning,” “glioma,” and “radiomics.” Five retrospective designed original articles including LGG and HGG subjects were chosen. Pooled sensitivity, specificity, their 95% confidence interval, area under curve (AUC), and hierarchical summary receiver-operating characteristic (HSROC) models were obtained.

**Result:**

The pooled sensitivity when diagnosing HGG was higher (96% (95% CI: 0.93, 0.98)) than the specificity when diagnosing LGG (90% (95% CI 0.85, 0.93)). Heterogeneity was observed in both sensitivity and specificity. Metaregression confirmed the heterogeneity in sample sizes (*p*=0.05), imaging sequence types (*p*=0.02), and data sources (*p*=0.01), but not for the inclusion of the testing set (*p*=0.19), feature extraction number (*p*=0.36), and selection of feature number (*p*=0.18). The results of subgroup analysis indicate that sample sizes of more than 100 and feature selection numbers less than the total sample size positively affected the diagnostic performance in differentiating HGG from LGG.

**Conclusion:**

This study demonstrates the excellent diagnostic performance of ML-based radiomics in differentiating HGG from LGG.

## 1. Introduction

Glioma is the most common primary malignant brain tumor that accounts for 80% of malignancies [1], and 2% of all cancers in US adults [2]. According to the World Health Organization (WHO) classification [3], gliomas are subdivided into two groups based on their malignant status low-grade glioma (LGG) for grades I to II with focal symptoms and high-grade glioma (HGG) for III to IV with generalized symptoms. Grade IV tumors called glioblastoma (GBM) account for 54% of all gliomas [4], with a median survival rate of 15 months [5]. Treatment of gliomas is essential since there is an eventual progression from LGG to HGG due to gliomas' distinctive molecular and clinical features [6]. For targeted treatment that is individualized to specific changes in individual tumors, different treatments including a near-total resection, postsurgical radiation, or temozolomide combined with radiation must be considered depending on the glioma's grade [2]. Therefore, the classification of tumor levels is crucial for intraoperative decision-making.

Magnetic resonance imaging (MRI) has been utilized to classify gliomas noninvasively for histopathological purposes. Recent studies have demonstrated the feasibility of conventional MRI sequences, especially gadolinium-based contrast-enhanced T1-weighted imaging (T1-CE) [7] when grading gliomas. With technological developments, advanced MRI sequences also contribute to physiological and metabolic assessments when classifying gliomas, such as perfusion-weighted imaging (PWI) [8] and diffusion-weighted imaging (DWI) [9]. However, previous studies on grading gliomas were limited due to utilizing only a small number of parameters extracted from a single MRI sequence.

A capability of radiomics analysis, which maximizes the number of quantitative image features from digital images, has great potential for the assessment of tumor biology [10]. The vast quantities of radiomics data enable information to be extracted from the entire tumor. As a result, radiomics can overcome intratumoral heterogeneities in both the molecular and histopathological assessment of tumors using quantitative values [11] that contribute to evidence-based decision-making in oncology. The assessment of both mutation status and gene expression, such as O6-methylguanine-DNA-methyltransferase (MGMT) gene expression or isocitrate dehydrogenase (IDH) mutation, is essential for predicting therapeutic responses when treating gliomas. Radiomics has proven the potential for the genotype classification of prognostic factors to predict IDH status, 1/19q codeletion status, or MGMT methylation status [12–14] in glioma-related studies. However, the treatment of gliomas cannot be processed by genotype alone since IDH mutation or 1/19q codeletion status tends to be used for classifying grades II and III and MGMT promoter methylation for grade IV. Therefore, the histological grade should also be incorporated into the genotype classification of gliomas. The vast amount of quantitative image features, including first-, second-, and higher-order statistical features, can represent histological values that include intensity differences and spatial interrelationships. As a result, radiomic features can provide distinctive information about tumor phenotypes and their microenvironments. Considering the heterogeneous histopathology biomarkers of angiogenesis, apoptosis, proliferation, and cellular invasion in gliomas [15], extracting a large amount of hidden data using radiomics could be a potential tool in classifying gliomas from single- or multiparameter MRI sequences.

As far as we know, no previous research has performed a systematic evaluation of the accuracy of machine learning- (ML-) based radiomics analysis in differentiating HGG from LGG. Therefore, the purpose of the study was twofold: first, to estimate the diagnostic accuracy of ML-based radiomics analysis in classifying HGG and second, to identify the potential covariates that could affect the diagnostic accuracy of ML-based radiomics.

## 2. Method

A meta-analysis was performed using Meta-DiSc version 1.4 (Unit of Clinical Biostatistics Team, Hospital Universitario Ramón y Cajal, Madrid, Spain). However, the Meta-DiSc version 1.4 uses outdated statistical methods since the Moses–Littenberg method does not account for between-study variances [16]. Therefore, RStudio (version 4.0.2) using the MADA package was implemented to utilize hierarchical summary receiver-operating characteristic (HSROC) models and bivariate models.

### 2.1. Literature Search

This meta-analysis was performed following the Preferred Reporting Items for Systematic Reviews and Meta-Analysis guidelines [17]. A primary literature search of the PubMed database was conducted to find all related literature in English between January 1, 2009, and May 1, 2020, including Medical Subject Headings (MeSH) and non-MeSH terms (see Supplementary Materials for key terms (available (here))).

### 2.2. Inclusion and Exclusion Criteria

All studies were selected by the following criteria: (a) original research articles; (b) patients with histopathologically confirmed WHO grade gliomas including both lower-grade glioma and high-grade glioma; (c) ML-based with radiomics features that were applied to classify gliomas using radiomics features; and (d) information for reconstructing 2 × 2 tables to estimate the diagnostic sensitivity and specificity for grading gliomas was included.

Studies were excluded if (a) they did not use ML to classify the grade of gliomas; (b) did not focus on differentiating between LGG and HGG; (c) no replies were received from the authors after requesting the data related to reconstructing the 2 × 2 table or subgroup analysis; and (d) they had a small sample size for performing the machine learning classifier.

### 2.3. Quality Assessment

Two independent reviewers conducted the quality assessment (S.C. and B.S.). Four main domains including patient selection, index test, reference standard, and flow and timing were evaluated based on the Quality Assessment of Diagnostic Accuracy Studies-2 (QUADAS-2) [18].

### 2.4. Statistical Analysis

A meta-analysis of the performance of radiomics-based ML in differentiating HGG from LGG was conducted. Therefore, the definition of true positive (TP) was set for HGG and true negative (TN) for LGG. For consistency, the TP definition in the study of Zhao et al. had to be redefined by switching sensitivity and specificity in the calculation. If the data for reconstructing the 2 × 2 table and analyzing subgroup analysis were insufficient, we contacted the authors.

The performance of the studies that implemented multiple ML classifiers for grading gliomas was averaged. For studies that reported the results of both the training and testing sets, the best result was selected for the meta-analysis. The performance of ML using radiomics in differentiating HGG from LGG was performed using the bivariate random effects model.

The following methods and criteria were used to estimate heterogeneity: (a) considering the small number of studies and lower statistical strength of Cochrane's *Q* test, *p* value <0.10 (not 0.05) indicated the presence of heterogeneity (14); (b) Higgins inconsistency index (*I*^2^) test values of 25%, 50%, and 75% defined the heterogeneity as low, moderate, or high; (c) a forest plot for assessing the heterogeneity in sensitivity and specificity and the visual assessment of the forest plots to assess the presence of threshold effect (increasing sensitivity with decreasing specificity and a positive correlation between sensitivity and the false-positive rate); (d) a large difference between the 95% confidence region and 95% prediction region in the HSROC curve; and (e) a Spearman correlation coefficient value >0.6, that indicated a threshold effect across all studies.

Metaregression and subgroup analysis were performed to explain the possible factors that contribute to heterogeneity and the factors contributing to the diagnostic performance of ML-based radiomics when grading gliomas with the following covariates: (a) subject number, (b) sequence types, (c) selected feature numbers, and (d) inclusion of the testing set.

## 3. Result

### 3.1. Literature Search

**Figure 1:**
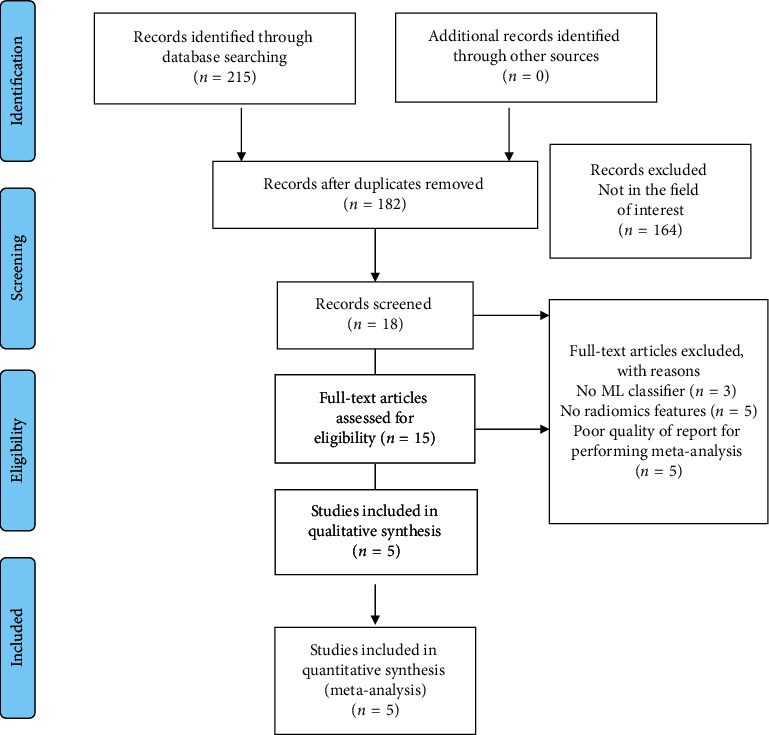
Search-strategy flowchart in accordance with the PRISMA guidelines used in the meta-analysis.

One hundred and ninety-four studies of interest were found, and five studies were selected for this meta-analysis after considering the inclusion and exclusion criteria (Figure 1).

### 3.2. Risk of Bias Assessment

Overall, a high risk of bias was estimated in the studies summarized in Table 1, with detailed descriptions given in Table 2. The high risk of bias could be attributed to the nature of the retrospective study, in which the patient's outcomes are known. Therefore, a case-control design for selecting patients already represents a high risk of bias. Furthermore, using public data could also lead to a high risk of bias in the QUADAS-2 assessment because not all acquisition factors for LGG and HGG in public data can be controlled. An assessor already knows the patients' diagnosis or reference standard results because WHO-classified patients were considered for research. ML-based radiomics analysis studies tend to find a methodological justification from a previous study method with an advanced ML classifier to improve the diagnostic accuracy. Therefore, prior knowledge before implementing the index test may introduce a high risk to the “index test” in the second domain. However, the reference standard for the histological diagnosis of HGG and LGG has already taken into account the accurate grading of gliomas that leads to reducing the risk of bias in the “reference standard” domain. Finally, though it was assumed that most were related to preoperative studies, it was unclear whether there was an appropriate interval between the index test and the reference standard or whether patients received a specific therapy. Therefore, the fourth domain of “flow and timing” in the reviewed studies had an unclear general bias content. Significant heterogeneity was present in data sources regarding image acquisition, feature engineering, and ad hoc analysis. Consequently, the quality assessment was limited regarding the applicability of ML-based radiomics analysis for grading gliomas.

### 3.3. Data Extraction

A summary of the results is presented in Table 3, while the method-related information is summarized in Table 4. Three of five studies utilized a single MRI sequence acquired by either conventional or advanced imaging [14, 19, 20], while the remaining studies implemented both conventional and advanced ASL and DWI sequences in [21, 22]. An imbalanced ratio was observed between the HGG and LGG datasets in the studies that used a large number of samples [19, 21], while the remaining three studies had a ratio equal to the sample [14, 20, 22]. Two of the five studies selected feature numbers equal to or greater than the total sample size [14, 20], while the remaining three studies selected fewer than the total sample size [19, 21, 22]. Only two studies included a testing set [20] without reporting the sample size of the testing set [19].

### 3.4. Heterogeneity Assessment

**Figure 2:**
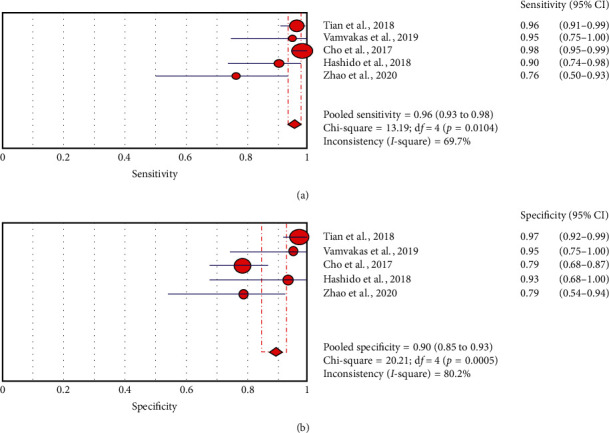
Pooled estimation of sensitivity and specificity for the diagnostic accuracy of radiomics using machine learning in differentiating HGG from LGG. Circles and horizontal lines represent the point estimate and 95% confidence intervals.

A forest plot was drawn to estimate the heterogeneity in sensitivity and specificity in Figure 2. Heterogeneity was found in both sensitivity (*I*^2^ = 69.70%, *p*=0.01) and specificity (*I*^2^ = 80.20%, *p* ≤ 0.01).

**Figure 3:**
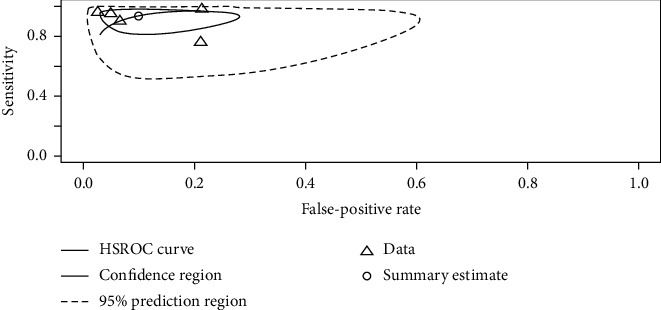
The HSROC curve displaying the diagnostic performance of ML-based radiomics in differentiating HGG from LGG. A large difference between the 95% confidence and prediction regions indicates a high possibility of heterogeneity across the reviewed studies.

A large difference between the confidence region and 95% prediction regions in the HSROC curve represents the possibility of heterogeneity across the studies in Figure 3.

### 3.5. Threshold Effect Assessment

The Spearman correlation coefficient between the sensitivity and false-positive rate was −0.4 (*p*=0.51), indicating the absence of a threshold effect. A threshold effect indicates a positive correlation between sensitivities and the false-positive rate that leads to a “shoulder arm” plot in the summary receiver-operating characteristic curve space. However, the visual assessment of the HSROC indicates the absence of a threshold effect as shoulder is absent in the HSROC space.

### 3.6. Data Analysis

Significant heterogeneity was observed in both pooled sensitivity (*I*^*2*^ = 69.70%, *p*=0.0104) and pooled specificity (*I*^*2*^ = 80.20%, *p* ≤ 0.01) as is shown in Figure 2. Therefore, the HSROC model based on a random effect model was applied to account for both intra- and interstudy variances in analyzing the diagnostic accuracy of the ML method with radiomics based on differentiating HGG from LGG. The area under the curve (AUC) value of 0.96 indicates high diagnostic performance.

### 3.7. Metaregression

A bivariate metaregression with a *p* value-based chi-squared statistic recommended by the Cochrane *diagnostic test accuracy (DTA) handbook* [23] was performed for the metaregression. As a result, the metaregression confirmed the heterogeneity in sample size (*p*=0.05), imaging sequence types (*p*=0.02), and data sources (*p*=0.01), but not in the testing set (*p*=0.19), feature extraction number (*p*=0.36), or selecting feature number (*p*=0.18). Bivariate metaregression based on the random effect model was also performed to analyze the regression coefficients between two groups related to four covariates: (a) subject number sample size of fewer than 100 vs. sample size of more than 100; (b) implementing only conventional or advanced MRI sequences vs. using both advanced MRI sequences and conventional MRI sequences; (c) selecting a feature number greater than or close to the total sample size vs. smaller than the total sample size; and (d) including testing set (validation set) or not.

The *z*-value for the regression coefficients for the sensitivities was significant for the sample size (*p* ≤ 0.01) and feature number (*p* ≤ 0.01). Therefore, the studies with sample sizes of more than 100 and a feature number smaller than the sample size exhibited better sensitivity, while the point estimate for the false positive rate did not indicate any effect. The *z*-value for the regression coefficient for the false-positive rate was significant (*p* ≤ 0.01) in the single-image sequences group, which offered a higher false-positive rate than the multiparameter image group. The point estimate for sensitivity did not indicate any effect. The *z*-values for the regression coefficient for both the sensitivity and false-positive rate were insignificant (*p*=0.3) and (*p*=0.4), indicating no statistical difference whether the testing set was included or not.

### 3.8. Subgroup Analysis

The sensitivity, specificity, positive likelihood ratio (PLR), negative likelihood ratio (NLR), and diagnostic odds ratio (DOR) were combined using a random effects model because of the heterogeneity across the reviewed studies in Table 5. In the subgroup analysis, the overall sensitivity of diagnosing HGG was higher (96% (95% CI, 0.93, 0.98)) than the specificity of diagnosing LGG (90% (95% CI, 0.85, 0.93)).

Similar to the result of metaregression, significant sensitivity, PLR, and NLR differences were found in the covariates of sample size and feature number; however, specificity was not detected. The studies using a sample size of more than 100 had higher sensitivity, PLR, and lower NLR than studies with a sample size of fewer than 100 (98 vs. 88), PLR (12.10 vs. 7.89), and NLR (0.03 vs. 0.14), but no difference in the specificity (90% vs. 90%). Furthermore, this result was aligned with the metaregression result, in which the studies using sample sizes of more than 100 had a lower false-positive rate.

In terms of feature engineering, the studies with selected feature numbers smaller than the total sample size had better sensitivity (97% vs. 85%), PLR (13.48 vs. 5.71), specificity (90% vs. 85%), and lower NLR (0.03 vs. 0.18) than the studies that selected feature numbers close to or greater than the total sample size. Furthermore, the lower false-positive rate result from the metaregression when selecting a number of features fewer than the total sample size was observed. However, there was no significant statistical difference in sensitivity between the studies that extracted only second-order features or both first- and second-order features (*p*=0.3), even with the higher sensitivity in the group that extracted both features (97% vs. 94%) in the subgroup analysis, but with lower specificity (84% vs. 95%), high PLR (11.19 vs. 7.23), and NLR (0.11 vs. 0.05).

No sensitivity difference was estimated in the group using single sequence and multiple sequences (96% vs. 96%), but the specificity was higher in the group using multiple sequences (97% vs. 81%) with a higher PLR (30.39 vs. 4.61) and lower NLR (0.04 vs. 0.09). Applying only the training set produced a higher sensitivity (94% vs. 97%), specificity (95% vs. 81%), PLR (12.91 vs. 5.32), and lower NLR (0.09 vs. 0.05) than the group that used both the training and testing sets. However, the result of metaregression indicated no statistical difference in either the sensitivity or false-positive rate. The overlap of the confidence interval in the sensitivity between the two groups was aligned with the result of metaregression. However, the higher specificity in the group that used only the training set indicated a better differentiation of LGG.

## 4. Discussion

Overall, the meta-analysis confirmed the source of heterogeneity from the covariates, including sensitivity, specificity, sample size, imaging sequences, and data source. The main reason for greater heterogeneity could be attributed to the nature of the multiple steps included in the radiomics process of image acquisition, data source, segmentation, feature engineering, and ad hoc analysis [24].

The results of the meta-analysis indicated that a greater than 100 sample size positively affected the diagnostic performance of sensitivity (HGG), but not specificity (LGG). This result could be attributed to two causes. First, having a large sample size is essential to improve training [25] and to avoid the overfitting that occurs in ML-based research. Therefore, both statistical analysis and ML training may favor the result of the group with a greater than 100 sample size. The total sample size difference between the two groups (507 vs. 122) also contributed to the results. Second, the result could be attributed mainly to the imbalance in the HGG and LGG sample ratio. This is the main challenge in the medical dataset, where the ML-based classification method prefers a larger to a smaller sample during training [26, 27]. Therefore, the imbalanced ratio between LGG and HGG (total sample 240 vs. 389) may increase the sensitivity due to the large sample size of HGG without considering the distribution of data ratio between LGG and HGG, while reducing the specificity performance [19, 21]. In contrast, the overall equal sample ratio was found in the group with a sample size of less than 100, as summarized in Table 3 [14, 20, 22]. As a result, the specificity may increase in the small sample size group that did not consider the LGG sample as a minor group during the classifier training. Therefore, there was no difference in the specificity even with the higher sample size of LGG in the large sample size group than in the small sample size group (117 vs. 54). Both limited and imbalanced numbers of samples between LGG and HGG across the studies also affected the separation of the validation and testing datasets. The majority of studies did not include the testing set because of the small sample size. Furthermore, poor-quality reporting that did not include the subject number used in training and testing [19] and included only four subjects in the testing set [20] was observed. Therefore, subgroup analysis that includes the testing set may not be an appropriate criterion for this meta-analysis, even though it is necessary for the external validation of the model. However, a higher specificity was observed in the group that included only the training set [14, 21, 22]. This could be attributed to the balanced ratio between LGG and HGG in the group described in Table 3, not to the inclusion of the testing set. In contrast, imbalanced ratios of large HGG samples over LGG samples were observed in the group that included both the training and testing sets [19, 20]. In short, it is reasonable to assume that the labeled data balance plays a key role in grading gliomas.

Interestingly, there was a significant difference in the specificity but not in the sensitivity between the groups using a single MRI sequence [14, 19, 20] and multiparametric images [21, 22]. The nonsignificant sensitivity difference between the two groups can be attributed to two reasons. First, quantifying the heterogeneous spatial gray distribution including intratumoral spatial variation and intensity of the entire tumor in the second-order features allowed for the classification of the heterogeneous HGG over the first-order statistical TA [21, 28]. Therefore, the second-order features that were extracted in all reviewed studies could contribute to a statistically insignificant sensitivity difference between the two groups. Second, the performance of combining different MRI techniques in differentiating HGG from LGG is questionable because the combination of conventional and advanced MRI sequences, including PWI, DWI, and magnetic resonance spectroscopy, did not significantly increase the glioma grading performance [29, 30]. Furthermore, conventional MRI variables including enhancement and necrosis have been reported as the major predictors in differentiating HGG from LGG above the combination of conventional MRI with PWI, DWI, and MRS [31]. Therefore, the sequence differences between the two groups may be insignificant because of the contributory role played by the conventional MRI sequences that were included in each group. As a result, no significant difference in the sensitivity between the single MRI sequence and the multiparametric image was observed in this study.

The higher specificity in the multiparametric group can be attributed to the apparent diffusion coefficient (ADC) values extracted from DWI [21] and cerebral blood flow extracted by perfusion imaging [22]. Even though the ADC values in differentiating LGG from HGG varied from study to study [29], T1-CE, ADC slow, T2 WI, and CBF have been widely used to classify LGG. Among various biomarkers, the ADC value has demonstrated the feasibility of classifying LGG by the higher ADC values in LGG than HGG [32, 33], and the entropy of ADC values among various texture analysis software showed promising results [7, 9]. Therefore, it is reasonable to assume that advanced MRI sequences for estimating angiogenesis and blood perfusion play a role in classifying LGG. In short, the higher false-positive rate in the single-image sequence group and the higher specificity of differentiating LGG in the multiple sequences resulted from the use of multiple imaging sequences for grading gliomas.

All results including sensitivity, specificity, PLR, and NLR were higher in the group with fewer feature numbers [14, 19, 21] than those that were greater than or equal to the total sample size [20, 22]. This can be attributed to two causes. First, the sample size difference between the two groups may affect the performance of the group that selected fewer feature numbers than the total sample size (547 vs. 82), as described in the following section of this paper. Second, selecting a method to reduce the dimensionality of feature space in radiomics is an essential component of feature selection. In fact, reducing redundant features is important to avoid overfitting and improving data, even though there is no gold standard for the appropriate number of features. Therefore, a small number of features are recommended, either one-tenth of the total sample data [34] or the square root of the total sample data [35]. Furthermore, a high contribution of the gray-level gradient cooccurrence matrix (GLGCM) features when grading gliomas suggested the importance of the second-order feature numbers in the study [21]. In short, the group that selected a smaller number of quantitative features than the total sample size exhibited better performance in grading gliomas.

### 4.1. Limitation

Several limitations were observed in our study. Only five studies were included in the meta-analysis, while the recommended number of studies for meta-analysis, according to the Cochrane DTA handbook, is at least 30 for sufficient power [23].

Therefore, the assessment of a publication bias using the Deek funnel plot asymmetry test was excluded because the small number of meta-analyses could skew the result due to the number difference between the small and large studies [36, 37] and the heterogeneous sources in the meta-analysis [38].

The poor quality of the report that excluded crucial result information contributed to the scarcity of literature for performing a meta-analysis of the accuracy of this diagnostic test in radiomics studies. Overall, the poor quality of the reporting has limited the study of radiomics in neurooncology [39] because guidelines for reporting quantitative imaging results have not yet been established. Regardless of the scarcity of literature, the total sample size of 629 subjects may be sufficient to represent the predictive value of ML-based radiomics analysis in differentiating HGG from LGG.

### 4.2. Future

Several factors should be improved for future studies related to ML-based radiomics for grading glioma. First, all reviewed studies did not include the updated WHO 2016 glioma classification standard for combining molecular profiling with histopathological profiling. Second, the dataset size still plays a key role in grading gliomas. Therefore, enlarging the dataset should also be considered to overcome the imbalance caused by oversampling a small sample to improve classifier performance [21].

Furthermore, the enlarged dataset would lead to separate training and testing sets that allow for the external validation of ML classifier performance. Third, incorporating patient demographics or clinical history should be considered to improve the classification of ML models. Finally, the variation process included in the radiomics analysis that is based on the numerical extraction approach to image analysis could affect the result due to bias and variance, not underlying biologic effects. Therefore, standardization in image acquisition, segmentation, feature engineering, statistical analysis, and the reporting format should be established for reproducibility and the generalization of ML-based radiomics studies. Essential steps for standardization include optimizing the standard imaging acquisition process, fully automating the process for segmentation and feature engineering, reducing the redundancy of feature numbers, enhancing the reproducibility of radiomics features, and transparently reporting results. Therefore, the following guidelines suggested by the relevant professional societies, such as the Society of Nuclear Medicine and Molecular Imaging, the Quantitative Imaging Network, Radiology Society of North America, and the European Society of Radiology that lead the field in imaging methods, including radiomics, should be considered. Furthermore, it has been reported that the magnet strength, flip angles, number of excitations, and different scanner platforms could affect both first-order and second-order features [40]. For example, the gray-level gradient cooccurrence matrix (GLCM) could be invariant to magnetic strength but susceptible to flip angles. However, the first-order features of entropy that are considered the most stable features have high reproducibility [41]. Therefore, the reproducibility of information related to radiomics features should be considered depending on the image acquisition method. Apart from the radiomics feature type, a segmentation method should be considered for the reproducibility of radiomics features. A registration distortion between MRI sequences could cause the incorrect localization of the region of interest and could affect the feature extraction process in radiomics analysis [42]. To reduce individual variability, deep learning-based automated segmentation and feature engineering gained attention [43]. Therefore, a deep learning-based approach should also be considered in radiomics analysis. The SVM-based classifier with recursive feature elimination (RFE) was found to be superior to other 25 ML-based classifiers and 8 independent attribute selection methods in grading gliomas using the multiparameter approach [44]. Therefore, an SVM-based classifier with RFE could be the best method to reduce feature redundancy and improve the ML classifier performance of glioma grading [28].

## 5. Conclusion

ML with radiomics demonstrated excellent diagnostic performance in differentiating HGG from LGG. The results of the meta-analysis provide the following recommendations to perform grading gliomas with ML-based radiomics: (a) use a large sample with oversampling of a minor class to balance the sample ratio and include the external validation set; (b) employ a multiparameter approach to extract the second-order features from the T1-CE sequence and ADC entropy from DWI; (c) select features with a number smaller than the total sample size by combining clinical information; and (d) implement the SVM classifier with SVM-REF attribute selection.

We submit that a methodological standard to ensure the reproducibility of ML-based radiomics is warranted for clinical application.

## Figures and Tables

**Table 1:** Summary of QUADS-2 tool assessment of the literature used in the meta-analysis.

Authors and year	Risk of bias			
	Patient selection	Index test	Reference standard	Flow and timing
Cho et al. 2018	+	+	−	?
Tian et al. 2018	+	+	−	−
Hashido et al. 2018	+	−	−	?
Vamvakas et al. 2019	+	+	+	?
Zhao et al. 2020	+	−	−	?

Risk of bias: + = high, − = low, and ? = unclear risk.

**Table 2:** Detailed QUADS-2 tool assessment after the two reviewers reached a consensus.

Authors and year	Patient selection	Index test	Reference standard	Flow and timing
Cho et al. 2018	The experiment was designed to be a retrospective study and did not include a random sample. However, the study avoided inappropriate exclusion (high risk)	The index test results were interpreted knowing the results of the reference standard. It was unclear whether a prespecified threshold was used (high risk)	It was unclear whether the reference standard was likely to classify the target condition accurately. The reference standard results were interpreted knowing the results of the index test (low risk)	It was unclear whether there was an appropriate interval between the index test and reference standard and whether all patients were included in the analysis. All patients received a reference standard, but it is unclear whether it was the same reference standard (unclear)

Tian et al. 2018	The experiment was designed to be a retrospective study and did not include a random sample. However, the study avoided inappropriate exclusion (high risk)	The index test results were interpreted knowing the results of the reference standard. However, a pre specified threshold was used (high risk)	The reference standard was likely to classify the target condition accurately. The reference standard results were interpreted without knowing the results of the index test (low risk)	It was clear whether there was an appropriate interval between the index test and reference standard. All patients received a reference standard, but it was unclear whether it was the same reference standard. Not all patients were included in the analysis (low risk)

Hashido et al. 2018	The experiment was designed to be a retrospective study and did not include a random sample. However, the study avoided inappropriate exclusion (high risk)	The index test results were interpreted without knowing the results of the reference standard. Furthermore, a pre specified threshold was used (low risk)	The reference standard was likely to classify the target condition accurately. The reference standard results were interpreted without knowing the results of the index test (low risk)	It was unclear whether there was an appropriate interval between the index test and reference standard. All patients received a reference standard, but it was unclear whether it was the same reference standard. Not all patients were included in the analysis (unclear)

Vamvakas et al. 2019	The experiment was designed to be a retrospective study and did not include a random sample. In addition, it was unclear whether the study avoided inappropriate exclusion (high risk)	The index test results were interpreted knowing the results of the reference standard. It was unclear whether a prespecified threshold was used (high risk)	The reference standard was likely to classify the target condition accurately. The reference standard results were interpreted knowing the results of the index test (high risk)	It was unclear whether there was an appropriate interval between the index test and reference standard and included all patients in the analysis. All patients received a reference standard, but it was unclear whether it was the same reference standard (unclear)

Zhao et al. 2020	The experiment was designed to be a retrospective study and did not include a random sample. In addition, it was unclear whether the study avoided inappropriate exclusion (high risk)	The index test results were interpreted without knowing the results of the reference standard. However, it was unclear whether a prespecified threshold was used (low risk)	It was unclear whether the reference standard was likely to classify the target condition accurately. The reference standard results were interpreted without knowing the results of the index test (low risk)	It was unclear whether there was an appropriate interval between the index test and reference standard. All patients received a reference standard, but it was unclear whether it was the same reference standard. Not all patients were included in the analysis (unclear)

**Table 3:** Summary of the results evaluated in the reviewed studies.

Study and year	Method	Algorithm	Dataset/HGG-LGG	MRI sequence	Best performance				Limitation
					AUC	DA (%)	Sen (%)	Spe (%)	
Cho et al. 2018	Classic machine learning	Multiple algorithms	WHO II–IV (*n* = 285)/210-75	T1, T1-C, T2, T2- FLAIR	0.94	92.92	97.86	79.11	No dataset separation information for training and testing cohort. Sample imbalance size between LGG and HGG.

Tian et al. 2018	Classic machine learning	SVM	WHO II–IV gliomas (*n* = 153)/111-42	Multiparametric	0.99	96.80	96.40	97.30	Sample imbalance sample size between LGG and HGG.

Hashido et al. 2018	Classic machine learning	Logistic regression	WHO II–IV (*n* = 46)/31-15	ASL, PWI (DSC)	0.96	NA	89.30	92.90	Small sample size. Small sample size used in the training set. Large feature number than the total sample size.

Vamvakas et al. 2019	Classic machine learning	SVM	WHO I–IV (*n* = 40) 20-20	Multiparametric	0.96	95.50	95	96	Small sample size.

Zhao et al. 2020	Classic machine learning	RF	WHO II-III gliomas (*n* = 36) 17-19	T1-C, T2- FLAIR	0.86	78.10	78.30	77.80	Small sample size. Large feature number compared to the total sample size.

**Table 4:** Summary of the methods used in the reviewed studies.

Study and year	Data source	External validation	Feature type	Feature extraction	Feature selection	Segmentation
Cho et al. 2018	Public	Training + testing	First-order and second-order (GLCM, ISZ)	486	5	ROI
Tian et al. 2018	Private	Training	First-order, second-order (GLCM, GLCGM)	510	30	VOI
Hashido et al. 2018	Private	Training (42) + testing (4)	First-order, second-order (GLCM, GLDM, GLRLM, GLSZM, and NGTDM)	91	75	Random forest-based semiautomatic tumor segmentation
Vamvakas et al. 2019	Private	Training	First-order and second-order texture (GLCM, GLRLM)	581	21	VOI
Zhao et al. 2020	Private	Training	First-order and second-order (GLCM, GLRLM, GLSZM, and GLDM)	1072	30	VOI

**Table 5:** Result of multiple subgroup analysis of machine learning-based radiomics for grading gliomas.

Subgroup	Study number	Patient number	Sensitivity	Specificity	PLR	NLR	Diagnostic odds ratio
All combined	5	629	0.96 (0.93–0.98)	0.90 (0.85–0.93)	9.53 (3.55–25.57)	0.07 (0.02–0.20)	153.85 (32.36–731.44)

*Populations*							
>100	2	507	0.98 (0.95–0.99)	0.90 (0.85–0.94)	12.099 (1.37–107.12)	0.03 (0.02–0.06)	393.81 (80.89–1917.3)_
<100	3	122	0.88 (0.78–0.95)	0.90 (0.77–0.96)	7.89 (2.21–28.15)	0.14 (0.05–0.39)	65.13 (7.84–540.95)

*Sequence*							
Single (CS or advanced)	2	262	0.96 (0.93–0.98)	0.81 (0.72–0.88)	4.61 (3.14–6.77)	0.09 (0.02–0.44)	66.75 (10.33–431.19)
Multiple (CS and advanced)	3	367	0.96 (0.91–0.99)	0.97 (0.92–0.99)	30.391 (11.585–79.726)	0.04 (0.017–0.09)	774.25 (202.54–2959.77)

*Feature number*							
≥Sample size	2	82	0.85 (0.72–0.94)	0.85 (0.69–0.95)	5.71 (1.39–23.46)	0.18 (0.07–0.52)	33.76 (3.36–339.14)
<Sample size	3	547	0.97 (0.95–0.99)	0.90 (0.85–0.94)	13.48 (2.56–71.12)	0.03 (0.02–0.06)	369.98 (19.68–6956.0)

*Training and testing set*							
Training set	3	331	0.94 (0.88–0.97)	0.95 (0.89–0.98)	12.91 (2.02–82.22)	0.09 (0.02–0.47)	154.56 (7.30–3276.9)
Training + testing set	2	298	0.97 (0.94–0.99)	0.81 (0.72–0.89)	5.32 (2.55–11.09)	0.05 (0.1–0.22)	176.99 (63.76–491.30)

## Data Availability

The data used to support the findings of this study are available upon request to skgtohn@ucl.ac.uk.

## Abbreviations

ASL: Arterial spin labeling
HGG: High-grade glioma
LGG: Low-grade glioma
ML: Machine learning
ADC: Apparent diffusion coefficient
FA: Fractional anisotropy
MRI: Magnetic resonance imaging
DWI: Diffusion-weighted imaging
DTI: Diffusion tensor imaging
GBM: Glioblastoma
SVM: Support vector machine
SVM-RFE: Recursive feature elimination
IDH: Isocitrate dehydrogenase
MGMT: O6-Methylguanine-DNA-methyltransferase
HSROC: Hierarchical summary receiver-operating characteristic curve
AUC: Area under curve
QUADAS-2: Quality assessment of diagnostic accuracy studies-2
TP: True-positive
TN: True-negative
PLR: Positive likelihood ratio
NLR: Negative likelihood ratio
DOR: Diagnostic odds ratio
RFE: Recursive feature elimination
GLCM: The gray-level gradient cooccurrence matrix.

## Additional Points

*Key Findings*. (i) Machine learning with radiomics demonstrated excellent diagnostic performance in differentiating high-grade glioma from low-grade glioma. (ii) The lower false-positive rate and higher specificity for classifying lower-grade glioma favored the use of multiparametric imaging, even though a nonstatistically significant sensitivity difference between the single-image sequence and multiparameter imaging group was observed. (iii) Based on the results of the meta-analysis, several factors positively affected the performance of machine learning-based radiomics when grading gliomas: (a) a large sample size with oversampling of a minor class to balance the sample ratio; (b) use of the multiparametric approach to extract the second-order features from the gadolinium-based contrast-enhanced sequence and an apparent diffusion coefficient entropy from diffusion-weighted image; (c) selection of features with fewer numbers than the total sample size combined with clinical information; and (d) implementation of a support vector machine classifier with support vector machine-recursive feature elimination attribute selection. (iv) A methodological standard to ensure the reproducibility of ML-based radiomics is warranted for clinical application.

## Supplementary Materials

Supplementary Materials The supplementary file contains the supplementary information for key terms.
