# Supplementary material for: Diagnostic Accuracy of Machine Learning-Based Radiomics in Grading Gliomas: Systematic Review and Meta-Analysis
Source: Contrast Media Mol Imaging. 2020 Dec 18;2020:2127062. doi: 10.1155/2020/2127062 (PMC7952179; doi:10.1155/2020/2127062)
Supplement: Supplementary Materials — The supplementary file contains the supplementary information for key terms. [file 2127062.f1.docx]

**Supplementary Information**

((((((("glioma"[MeSH Terms] OR "brain neoplasms"[MeSH Terms] OR "glioblastoma"[MeSH Terms] OR "classification"[MeSH Terms]) AND "machine learning"[MeSH Terms]) OR "artificial intelligence"[MeSH Terms] OR "support vector machine"[MeSH Terms]) AND ("radiomic"[All Fields] OR "radiomics"[All Fields]) AND 2009/01/01:2020/05/01[Date - MeSH]) NOT "comment"[Publication Type]) NOT "editorial"[Publication Type]) NOT "letter"[Publication Type]) NOT "meta-analysis"[Publication Type]

- Keywords used in the PubMed database to find all related literature in English between January 1, 2009, and May 1, 2020 including Medical Subject Headings (MeSH) and non-MeSH terms
